# Characterization of the intrinsic AmpC β-lactamase of *Escherichia marmotae* and its impact on decreased susceptibility to cefiderocol and resistance to broad-spectrum cephalosporins

**DOI:** 10.1093/jac/dkag271

**Published:** 2026-08-13

**Authors:** Samanta Freire, Sandra Martinez Alvarez, Jacqueline Findlay, Miriam Vogel, Patrice Nordmann, Laurent Poirel

**Affiliations:** Emerging Antibiotic Resistance, Medical and Molecular Microbiology, Faculty of Science and Medicine, University of Fribourg, Fribourg, Switzerland; Emerging Antibiotic Resistance, Medical and Molecular Microbiology, Faculty of Science and Medicine, University of Fribourg, Fribourg, Switzerland; Emerging Antibiotic Resistance, Medical and Molecular Microbiology, Faculty of Science and Medicine, University of Fribourg, Fribourg, Switzerland; MCL, Medizinische Laboratorien, Niederwangen, Switzerland; Emerging Antibiotic Resistance, Medical and Molecular Microbiology, Faculty of Science and Medicine, University of Fribourg, Fribourg, Switzerland; Swiss National Reference Center for Emerging Antibiotic Resistance, Fribourg, Switzerland; Emerging Antibiotic Resistance, Medical and Molecular Microbiology, Faculty of Science and Medicine, University of Fribourg, Fribourg, Switzerland; Swiss National Reference Center for Emerging Antibiotic Resistance, Fribourg, Switzerland

## Abstract

**Objectives:**

We aimed to characterize the intrinsic AmpC β-lactamase (EMC-2) from an *Escherichia marmotae* clinical isolate recovered from a bloodstream infection, exhibiting resistance to broad-spectrum cephalosporins.

**Methods:**

Cloning experiments in *Escherichia coli* were performed to evaluate the impact of the respective AmpC enzymes on β-lactam susceptibility. Purification of the enzymes was performed by ion-exchange chromatography, and kinetic assays were performed by UV spectrophotometry.

**Results:**

By comparing the sequence of this AmpC with that of a WT *E. marmotae*, four amino acid substitutions and one amino acid deletion at position 296 were identified. Furthermore, a promoter alteration was responsible for an enhanced gene expression. Production of EMC-2 in *E. coli* TOP10 conferred an extended-spectrum AmpC (ESAC) phenotype characterized by increased MICs of cefotaxime, cefepime and cefiderocol. Site-directed mutagenesis confirmed the important role of the Arg296 deletion in this phenotype.

**Conclusions:**

We showed that the intrinsic AmpC of *E. marmotae* can acquire both structural and regulatory modifications that broaden its substrate spectrum, leading to reduced susceptibility to clinically relevant β-lactams, including cefiderocol.

## Introduction

*Escherichia marmotae* is a Gram-negative bacterium belonging to the Enterobacterales order and is phylogenetically closely related to *Escherichia coli*. Initially isolated and identified in 2015 from wild Himalayan marmots,^1^ it has recently been associated with invasive human infections.^2^ Similarly to *E. coli*, *E. marmotae* naturally possesses a chromosomally located AmpC cephalosporinase gene, which is usually expressed at a very low level due to a weak promoter, explaining why isolates belonging to that species are subsequently fully susceptible to all β-lactams.^3^ However, it has been reported that some *E. marmotae* are resistant to β-lactams, as a consequence of overexpression of this AmpC-encoding gene or consequent to plasmid-mediated β-lactamase gene acquisitions, including ESBLs and carbapenemases.^2^

## Methods

*E. marmotae* strain N4973 was isolated from a blood culture of a 63-year-old female patient hospitalized at the ICU of a Swiss hospital due to complications of an adenocarcinoma of the caecum and the endometrium. From 2 January 2024 she had received meropenem 1 g/8 h over 8 days. On 31 January the *E. marmotae* strain was recovered and the patient was successfully treated with cefepime 1 g/24 h over 3 days. The species was identified by MALDI-TOF. Susceptibility testing was performed by disc diffusion and broth microdilution and interpreted according to EUCAST guidelines (https://www.eucast.org/fileadmin/src/media/PDFs/EUCAST_files/Breakpoint_tables/v_15.0_Breakpoint_Tables.pdf).

## Results and discussion

N4973 was resistant to broad-spectrum cephalosporins, including ceftazidime and cefepime, and additionally showed reduced susceptibility to both ceftazidime/avibactam (4 mg/L) and cefiderocol (2 mg/L) (Table 1). Disc diffusion testing in the presence of 250 mg/L cloxacillin showed restoration of inhibitory activity for broad-spectrum cephalosporins, suggesting the production of an AmpC-type enzyme. PCR screening of acquired AmpC-encoding genes was performed, using primers targeting the following β-lactamase genes: *bla*_CMY_, *bla*_DHA_, *bla*_ACC_, *bla*_ACT_, *bla*_LAT_, *bla*_FOX_, *bla*_MOX_ and *bla*_EBC_; this yielded negative results. Therefore, it was speculated that this might correspond to an intrinsic AmpC production in that species. A search performed over the Genbank databases revealed that an *ampC* gene was indeed present in the chromosome of *E. marmotae* reference strain DSMZ 28771, the corresponding protein sharing significant identity with the intrinsic AmpC of *E. coli*, differing by 20–30 amino acids out of the total 370 amino acid sequence, depending on the *E. coli* genomes considered in the databases. To allow for further genotypic characterization, N4973 was then subject to Illumina WGS, and subsequent analyses were performed using the Center for Genomic Epidemiology Platform (https://www.genomicepidemiology.org/). N4973 did not harbour any β-lactamase genes, aside from the chromosomally encoded AmpC, and the *E. marmotae* species identification was confirmed using Kmer Finder.^4^

**Table 1.** MICs for the clinical isolate, recombinant strains producing EMC-2 and EMC-1, and the mutated R296-deleted EMC-1

	MIC, mg/L									
Isolate	AMX	FOX	CTX	CAZ	CZA	FEP	ETP	IPM	MEM	FDC
N4973	>128	32	2	64	4	2	≤0.125	≤0.125	≤0.125	2
DSMZ 28771	4	8	≤0.125	0.5	≤0.125	≤0.125	≤0.125	≤0.125	≤0.125	≤0.125
TOP10/pEMC-2	>128	128	16	128	8	32	≤0.125	≤0.125	≤0.125	2
TOP10/pEMC-1	>128	32	2	4	0.25	≤0.125	≤0.125	≤0.125	≤0.125	≤0.125
TOP10/EMC-1_R296del	>128	8	8	64	2	8	≤0.125	≤0.125	≤0.125	2

AMX, amoxicillin; CAZ, ceftazidime; CTX, cefotaxime; CZA, ceftazidime/avibactam; ETP, ertapenem; FDC, cefiderocol; FEP, cefepime; FOX, cefoxitin; IPM, imipenem; MEM, meropenem.

The DSMZ 28771 reference strain was obtained and tested for susceptibility to β-lactams, and was found to exhibit full susceptibility, suggesting either a lack of expression of the *ampC* gene or a defective β-lactamase activity of the corresponding enzyme. Then, from the database sequences, primers ampC-EmF (5′-GTTTTCTACGGTCTGGCTGC-3′) and ampC-EmR (5′-AATTCGGGCCATCTTCGTTG-3′) were designed to amplify the whole *ampC* gene from reference strain DSMZ 28771 and from isolate N4973, respectively. The amplified genes were then subjected to Sanger sequencing and cloned into the plasmid pCR-Blunt II-TOPO (Invitrogen, Thermo Fisher, Basel, Switzerland) and subsequently transformed into *E. coli* TOP10. Analysis of the *ampC* gene sequences of the reference strain and the N4973 isolate respectively identified four amino acid differences between the two corresponding protein sequences (respectively named EMC-1 and EMC-2, standing for ***E****. **M**armotae* Amp**C**), resulting in amino acid substitutions Thr17Asp, Thr77Ala and Thr310Ala, together with a deletion of the Arg amino acid at position 296. Of note, position 296 is located within the enzyme’s R2 loop, suggesting a significant impact in terms of hydrolysis profile.^5^ Indeed, structural modifications in the AmpC R2 loop have previously been identified as a key factor in the heightened resistance to broad-spectrum cephalosporins in *E. coli*.^6^

Susceptibility testing of both recombinant strains showed that the *E. coli* recombinant strain producing the EMC-2 variant exhibited significantly higher MICs not only of cefoxitin, cefotaxime, ceftazidime, cefepime and cefiderocol, but also of ceftazidime/avibactam, compared with the *E. coli* recombinant strain producing EMC-1. Owing to its capacity to impact susceptibility to cefepime and cefiderocol, EMC-2 was therefore defined as an extended-spectrum AmpC (ESAC).^7^

To decipher the extended spectrum of the EMC-2, site-directed mutagenesis using the Q5 Site-Directed Mutagenesis kit (ref. E0554S; New England Biolabs, Ipswich, MA, USA) was performed using EMC-1 as template, to generate deletion of the Arg296 residue. The presence of the deletion was confirmed by PCR amplifications of the EMC-2 and Sanger sequencing. Susceptibility testing performed in the generated mutant *E. coli* recombinant strain showed significantly increased MICs of cefotaxime, ceftazidime, cefepime, ceftazidime/avibactam and cefiderocol, thus indicating the critical role of this deletion in the ESAC phenotype. Notably, previous studies performed on the AmpC of *E. coli* showed that various mutations and/or deletions at position 296 had a significant impact on the increased resistance to broad-spectrum cephalosporins, highlighting this residue as a ‘hotspot’ for mutations leading to an ESAC phenotype.^7,8^

By analysing the sequences upstream of the *ampC* gene of *E. marmotae* N4973, a single nucleotide insertion was identified between positions −10 and −35 of the putative promoter sequence (by analogy with that of the well-characterized *ampC* of *E. coli*, but also comparing with the WT strain DSMZ 28771), enlarging the distance between the TTGTCA and TACAAT boxes from 16 to 17 nucleotides. Alterations affecting the distance between the −10 and −35 boxes in the *E. coli ampC* promoter have previously been shown to enhance the interaction between RNA polymerase and this region during the early stages of transcription,^9,10^ maximizing the promoter’s activity and resulting in overexpression of the AmpC-encoding gene. A previous study in *E. coli* identified that the exact promoter mutation observed in N4973 resulted in significant up-regulation of AmpC production, as assessed by proteomic analyses.^11^

Both EMC-1 and EMC-2 were purified from the respective *E. coli* TOP10 recombinant strains, using the previously described purification protocol.^9,12^ Kinetic measurements were then performed using cephalothin as a reporter substracte, at 262 nm and with a molar absorption coefficient (Δε)  of 7960 M^–1^.cm^−1^ , as previously described.^12^ Kinetic parameters revealed that both the EMC-1 and EMC-2 enzymes hydrolysed cephalothin significantly and at a similar level overall, even if significant discrepancies in terms of affinity for that substrate were observed (EMC-2: *k*_cat_ 7.8 s^−1^, *K*_m_ 14.2 µM, *k*_cat_/*K*_m_ 0.6 µM^−1^.s^−1^; EMC-1: *k*_cat_ 37 s^−1^, *K*_m_ 169 µM, *k*_cat_/*K*_m_ 0.2 µM^−1^.s^−1^). However, when testing other substrates, no significant hydrolysis of broad-spectrum cephalosporins was detected with either enzyme, therefore affinity constants (*K*_i_) were determined. It revealed very high affinities for ceftazidime (*K*_i_ 0.46 µM), cefepime (*K*_i_ 3.31 µM) and cefiderocol (*K*_i_ 0.10 µM) for EMC-2. By contrast, the affinity of EMC-1 for ceftazidime was moderate (*K*_i_ 47 µM) and no inhibition was observed for cefepime and cefiderocol.

### Conclusions

Despite infections caused by *E. marmotae* being rarely reported, and particularly with cephalosporin-resistant strains, it should be highlighted that misidentification at the species level may result in underestimations of both their prevalence and their resistance patterns.^2^ Here we showed that occurrence of intrinsic ESAC enzymes may be observed in this bacterial species, as previously shown in *E. coli*, *Serratia marcescens*, *Acinetobacter baumannii* or *Pseudomonas aeruginosa*.^13–16^ This study further highlights the impact of some intrinsic AmpC enzymes on resistance to broad-spectrum cephalosporins, when the corresponding gene actually combines specific substitutions in its coding sequence and possibly also in its promoter region. The specific genetic context evidenced here was indeed explaining both the hyperproduction and enlarged catalytic activity of EMC-2, leading to a reduced susceptibility to critical antibiotics of the clinical isolate, including cefepime, ceftazidime/avibactam and cefiderocol.

## Data Availability

The raw sequencing reads generated in this project are available at NCBI under BioProject accession number PRJNA1492588.
